# Innovations in bio-engineering and cell-based approaches to address immunological challenges in islet transplantation

**DOI:** 10.3389/fimmu.2024.1375177

**Published:** 2024-04-08

**Authors:** Beatrice Xuan Ho, Adrian Kee Keong Teo, Natasha Hui Jin Ng

**Affiliations:** ^1^ Stem Cells and Diabetes Laboratory, Institute of Molecular and Cell Biology (IMCB), Agency for Science, Technology and Research (A*STAR), Singapore, Singapore; ^2^ BetaLife Pte Ltd, Singapore, Singapore; ^3^ Department of Biochemistry, Yong Loo Lin School of Medicine, National University of Singapore, Singapore, Singapore; ^4^ Department of Medicine, Yong Loo Lin School of Medicine, National University of Singapore, Singapore, Singapore; ^5^ Precision Medicine Translational Research Programme, Yong Loo Lin School of Medicine, National University of Singapore, Singapore, Singapore

**Keywords:** stem cells, regenerative medicine, diabetes, islet cells, beta cells, islet transplantation, HLA, hypoimmune

## Abstract

Human allogeneic pancreatic islet transplantation is a life-changing treatment for patients with severe Type 1 Diabetes (T1D) who suffer from hypoglycemia unawareness and high risk of severe hypoglycemia. However, intensive immunosuppression is required to prevent immune rejection of the graft, that may in turn lead to undesirable side effects such as toxicity to the islet cells, kidney toxicity, occurrence of opportunistic infections, and malignancies. The shortage of cadaveric human islet donors further limits islet transplantation as a treatment option for widespread adoption. Alternatively, porcine islets have been considered as another source of insulin-secreting cells for transplantation in T1D patients, though xeno-transplants raise concerns over the risk of endogenous retrovirus transmission and immunological incompatibility. As a result, technological advancements have been made to protect transplanted islets from immune rejection and inflammation, ideally in the absence of chronic immunosuppression, to improve the outcomes and accessibility of allogeneic islet cell replacement therapies. These include the use of microencapsulation or macroencapsulation devices designed to provide an immunoprotective environment using a cell-impermeable layer, preventing immune cell attack of the transplanted cells. Other up and coming advancements are based on the use of stem cells as the starting source material for generating islet cells ‘on-demand’. These starting stem cell sources include human induced pluripotent stem cells (hiPSCs) that have been genetically engineered to avoid the host immune response, curated HLA-selected donor hiPSCs that can be matched with recipients within a given population, and multipotent stem cells with natural immune privilege properties. These strategies are developed to provide an immune-evasive cell resource for allogeneic cell therapy. This review will summarize the immunological challenges facing islet transplantation and highlight recent bio-engineering and cell-based approaches aimed at avoiding immune rejection, to improve the accessibility of islet cell therapy and enhance treatment outcomes. Better understanding of the different approaches and their limitations can guide future research endeavors towards developing more comprehensive and targeted strategies for creating a more tolerogenic microenvironment, and improve the effectiveness and sustainability of islet transplantation to benefit more patients.

## 1 Introduction

Diabetes is a chronic metabolic disorder that affects 537 million adults aged between 20 to 79 years old. Disease prevalence is increasing year on year and is predicted to reach 643 million by 2030 (1). Individuals with poorly controlled diabetes face increased risk of heart disease, kidney disease, nerve complications and eye disorders. Type 1 diabetes (T1D) constitutes 5% to 10% of all diabetes cases, whereas the more common Type 2 diabetes (T2D) accounts for majority of the remaining 90% to 95% of diagnosed cases (2). T1D is an autoimmune disease resulting from the body’s immune system attacking the insulin-producing β cells of the pancreatic islets (3). This irreversible loss of β cells results in insulin deficiency, impaired glucose uptake in the peripheral tissues, and consequently hyperglycemia. The early onset of T1D, often during adolescence, results in the need for life-long insulin therapy and intensive blood glucose monitoring to prevent both hyperglycemia and hypoglycemic episodes, which can severely impact the quality of life of patients (4). Approximately 25% of T1D patients additionally suffer from impaired awareness of hypoglycemia (IAH) (defined as the diminished ability to perceive the onset of low blood glucose levels), which is associated with elevated risk of severe hypoglycemic events (SHEs) and consequently higher risk of morbidity and mortality (5). Furthermore, the risk of hypoglycemic events increases with the duration of T1D.

On the other hand, T2D is a common chronic condition caused primarily by defective insulin secretion from the pancreatic β cells and/or insulin resistance (6). It has a multitude of risk factors including obesity, genetic predisposition, physical inactivity, diet contributions and ageing, and therefore has a wide range of treatment options from lifestyle intervention to oral medications and insulin therapy. A vicious cycle exists in which persistent hyperglycemia leads to progressive decline in β cell compensation and eventual onset of β cell dysfunction (7–9). As a result, subjects progress from normal glucose tolerance to impaired glucose tolerance, and ultimately develop full-fledged T2D.

Replacement of β cell function through pancreatic islet transplantation is an established standard of care procedure (akin to organ tissue transplant) to treat T1D patients with impaired hypoglycemia awareness and who experience multiple SHEs in several countries, such as Canada, Australia, parts of Europe and Asia (10). Human islets for allogeneic use are isolated from deceased donor pancreases, following a series of tissue digestion, isolation, purification, and qualification steps. As prescribed in the Edmonton Protocol, which played a key role in revolutionizing islet transplantation since the 2000s, human islets are transplanted by infusion into the hepatic portal vein of the recipient based on the required islet equivalents (IEQ) per kilogram of the recipient’s body weight, alongside a steroid-free immunosuppression regimen (11). Patients may need to be dosed with multiple islet infusions from different donor pancreases to achieve euglycemia successfully. The procedure has since remained the standard protocol for islet transplantation and was seen as a promising step towards a T1D cure. Long term follow ups of patients for up to 20 years after transplant at a single centre showed that those with sustained graft survival no longer suffered from SHEs, displayed better insulin independence, and long term safety despite chronic immunosuppression (12). Another 5 year follow up of over 1200 patients across multiple centres similarly established the overall safety and efficacy of islet transplantation (13). Patients with T1D benefit from allogeneic islet transplantation through substantial improvement in glycemic control, almost complete abrogation of SHEs, reduction in insulin doses, and ultimately improvement in quality of life.

While islet transplantation may be life-changing for T1D patients, patients need to be willing and able to undergo long term, intensive immunosuppression. As with other solid organ transplantation, the side effects and complications that result require careful consideration of the risk to benefit ratio. The treatment can result in serious side effects such as increased risk of infections, malignancies, kidney damage, vomiting, nausea and diarrhoea (14–16). Immediate complications associated with intrahepatic islet transplantation includes instant blood-mediated inflammatory reaction (IBMIR), caused by direct exposure of the islets to the bloodstream, which triggers pro-inflammatory cytokine release followed by complement activation and recruitment of innate immune cells which further exacerbates inflammation and destruction of islets (17). For these reasons, the overall impact of islet transplantation in its present form remains limited. With regards to T2D, due to the multifactorial nature of diabetes development and the presence of insulin resistance, patients with T2D have yet to be considered for islet cell replacement therapy in the clinical setting. However, it is possible to consider that specific subsets of T2D patients that have severe insulin deficiency with normal insulin sensitivity (18) may benefit from renewable islet cell replacement to reinstate insulin production.

With the increasing prevalence of T1D and T2D globally, patient eligibility issues and complications associated with immunosuppression, coupled with overall shortage of cadaveric human islets will aggravate the socio-economic burden from disease. This situation highlights the need for novel approaches to protect islet allografts and overcome immunological challenges associated with allogeneic islet transplantation. In this review, we will examine the current status of primary human islet transplantation, the key challenges surrounding the need to undergo chronic immunosuppression and the lack of sufficient human donor islets. We also touch on developments in transplantation of islet cells derived from alternative sources, and promising avenues using bio-engineering or cell-based engineering approaches to protect transplanted islets from immune rejection.

## 2 Human allogeneic primary islet transplantation and its associated challenges

### 2.1 Current status of pancreatic islet transplantation

Human pancreatic islet transplantation offers a functional source of β cells for the treatment of diabetes, especially in a subset of patients with T1D who are prone to hypoglycemic unawareness and experience severe hypoglycemia despite optimal management of glycemic levels (11, 12, 19, 20). These have far-reaching benefits beyond physiological changes such as improvement in patients’ mental health, relief for caregivers, resumption of work productivity and reduced ambulance conveyance and emergency care needed. The success of the treatment was made possible due to the seminal research by Shapiro and team, who developed the Edmonton Protocol, building on previous achievements by others (for a detailed review on the history of clinical islet transplantation please see (21)). The procedure recommends transplantation of a cumulative islet mass of at least 10,000 IEQ per kilogram of the recipient’s body weight. This typically required at least two infusions from different donor material, unless insulin independence was achieved with a single infusion (11, 20). Islets are transplanted into the hepatic portal vein, the current clinical gold standard route, avoiding the need for surgery. The procedure allows the islet cells to access the circulatory system and facilitate glucose sensing and insulin release into the bloodstream, effectively restoring glycemic control in patients. Repeated islet infusions do carry the risk of procedure-related bleeding arising from elevated intraportal vein pressure and portal vein thrombosis (22). The protocol had set a standard for the infusion of an adequate islet mass combined with a glucocorticoid-free immunosuppressive regimen (e.g tacrolimus and sirolimus) (20). In recent practices, daclizumab (non-depleting monoclonal anti-interleukin-2 receptor antibody) and/or anti-thymocyte globulin is administered as pre-procedural induction immunosuppression, whereas low-dose tacrolimus (calcineurin inhibitor) in combination with mycophenolate mofetil or sirolimus is prescribed for maintenance immunosuppression (23). Sirolimus (mTOR inhibitor) has been found to be more poorly tolerated by patients with adverse side effects, hence its exclusion may result in improved longer-term outcomes. Likewise, although tacrolimus-based immunosuppression is effective against allo- and auto-immune rejection, its side effects include nephrotoxicity and diabetogenicity due to effects on the islet cells. Additionally, other anti-inflammatory agents are needed in the peri-transplant periods to counter proinflammatory cytokines and preserve islet function, such as etanercept (TNFα blocker) and anakinra (IL-1 receptor antagonist) which were found to be associated with improved clinical outcomes as compared to regimens without the use of anti-inflammatory agents (12, 24). Thus, ongoing research efforts remain important to define immunosuppressant and anti-inflammatory drug combinations with better safety profiles while remaining effective for preserving islet graft function.

Islet transplantation has been and will continue to be a life-changing therapy as it has resulted in positive outcomes for patients including insulin independence, glycemic control, freedom from SHEs and restoration of hypoglycemia awareness. These outcomes are positively correlated with graft survival and function (fasting C-peptide >0.1 nmol/L post-transplantation) and achievement of HbA1c level of <7.0% (53 mmol/mol) at least 1 year post-transplant (12, 19, 25). Furthermore, despite the long period under an immunosuppressive regimen, sustained islet function in those with sustained graft survival is possible, though the incidence of cancer appeared to be higher (12, 19, 25). In the recently FDA-approved donor-derived pancreatic islet cell therapy for T1D, known as Lantidra or donislecel (manufactured by CellTrans Inc.), the therapy is indicated for adults with T1D where exogenous administration of insulin is insufficient to maintain the HbA1c target and who experience hypoglycemia unawareness (26, 27). FDA approval was based on experiences from Phase I/II clinical trials that demonstrated graft survival in all 10 patients and insulin independence maintained in 60% of patients 5 years post-transplant, as well as in another Phase III clinical trial revealing that all 21 patients were free from hypoglycemic episodes and most maintained HbA1c levels at ≤ 6.5% at a 1-year follow up (28, 29). Importantly, no significant side effects were reported for the cell therapy except for procedural-related bleeding. The approval represents a positive step forward for T1D management in the US, though there remained controversy over the recognition of islets as drugs instead of organs that may place a limitation over patient access.

### 2.2 Obstacles limiting widespread adoption of human primary allogeneic islet transplantation

**Figure 1:**
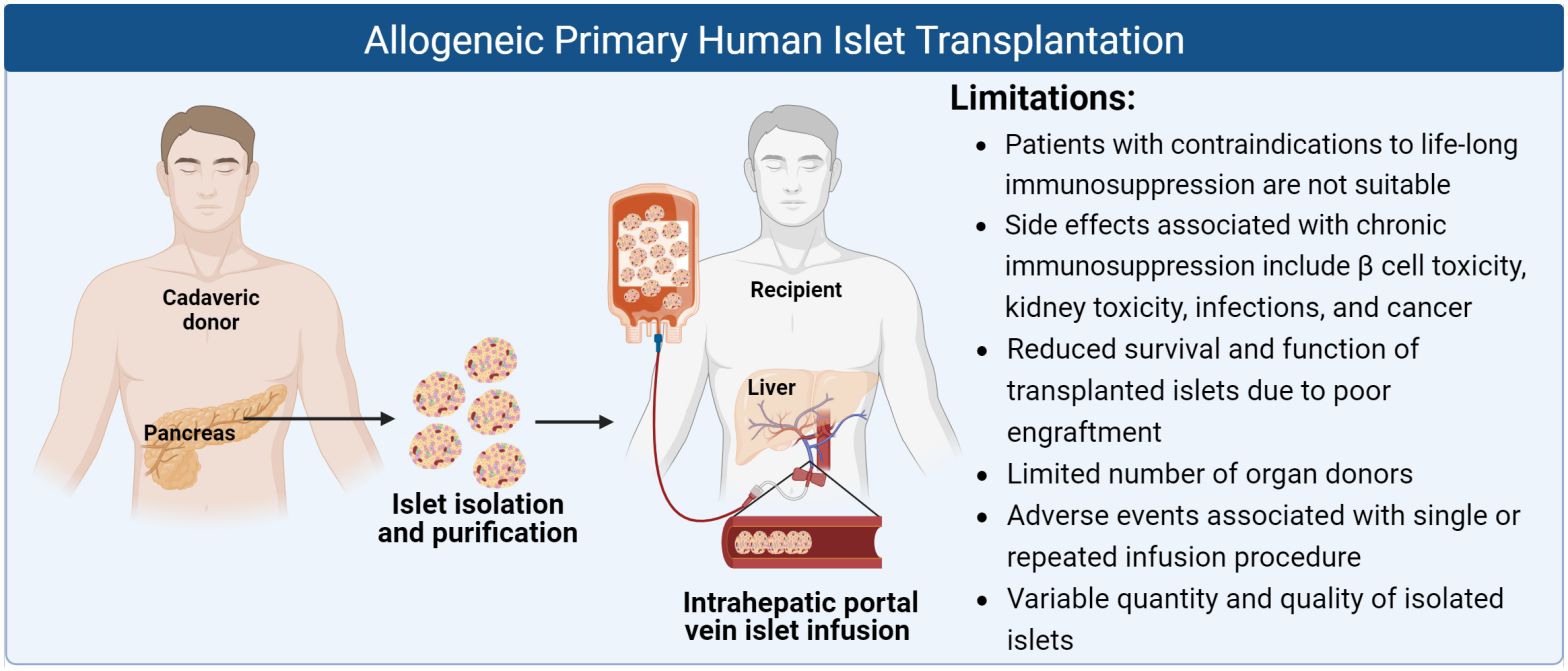
Schematic of allogeneic primary human islet transplantation and its limitations (Created with BioRender.com).

Islet transplantation has proven to be a promising curative approach for T1D patients, both saving lives and improving quality of life. However, there remain several challenges hindering its widespread clinical utility, particularly the need for chronic immunosuppressive therapy and limited donor islet availability (**Figure 1**).

Firstly, as islet transplant patients are required to undergo intensive life-long immunosuppression to prevent graft rejection and loss of islet function, the selection of immunosuppressants used may induce side effects or autoimmunity recurrence, which will influence islet transplantation outcome. For instance, induction of immunosuppression with anti-thymocyte globulin as compared to daclizumab, and maintenance of immunosuppression with tacrolimus as compared to sirolimus, has been shown to increase risk of autoantibody recurrence in islet transplantations (30). This study highlighted the “off-target” effects of immunosuppressants, particularly how immunosuppressants influence the profile of regulatory T cells (Tregs), which are an important subset of immunomodulatory T cells responsible for promoting immune tolerance. Immunosuppressants that foster a richer Tregs environment could drive tolerance and further minimize the need for immunosuppression (31). Previous studies investigating the impact of immune-modulatory drugs on the function of Tregs showed that sirolimus has a Tregs-favoring effect as compared to tacrolimus (32). Greater clarity on the immunological mechanisms mediated by the immunosuppressants would guide future directions in preserving Tregs numbers and function for better success in balancing immunosuppression and transplant outcome.

Chronic immunosuppression has also been associated with other detrimental side effects, such as β cell toxicity, kidney toxicity, higher risk of cancer and opportunistic infections as the protective function of the patient’s immune response is jeopardized. Reported symptoms experienced by patients include anaemia, nausea, fatigue, diarrhoea, and abdominal pain, though the incidents varied amongst patients and depended on the number of islet infusions and length of follow-up (33, 34). Common adverse effects occurred in between 20% to 90% of patients from initial infusion up to 1 year following final infusion, that are mostly related to the infusion procedure and immunosuppressive regimen administered (34). In the event of a life-threatening infection or cancer requiring discontinuation of immunosuppressive medications, there was eventual loss of islet cell function and resumption of insulin dependence (34). Additionally, patients who are contraindicated for immunosuppression, such as those with relevant drug allergies or who are highly susceptible to acute or chronic infections, are not eligible for islet transplant. T1D patients often possess pre-existing renal impairment due to longstanding diabetes and their renal dysfunction may be exacerbated by tacrolimus which can result in calcineurin-induced nephrotoxicity (35, 36). In addition to nephrotoxicity, tacrolimus is further associated with gastrointestinal side effects leading to episodic diarrhoea. Sirolimus has also been linked to several side effects including mouth ulcerations, neutropenia, dyslipidemia, small bowel ulceration, peripheral edema, and the development of ovarian cysts in females (37). The lifetime risk of lymphoma is estimated to be 1-2% in transplant recipients undergoing long-term immunosuppression, with the most common malignancies being non-melanomatous skin cancers (38). At supratherapeutic levels, tacrolimus and sirolimus have also been associated with human islet toxicity caused by increased amyloid deposition and disrupted insulin granule formation, though the detrimental effects on the β cells may be reversible upon withdrawal of drug treatment (39). For all these reasons, patients must be screened for endogenous infections or pre-existing medical conditions that can be aggravated following immunosuppressant therapy, and the risks weighed alongside the benefits (non-recurring severe hypoglycemia and achieved target HbA1c) to patients. Hence, islet transplantation has only been considered for T1D patients complicated by IAH and SHEs, when other lines of treatment have failed to prevent life-threatening SHEs, placing a significant limitation on the large pool of diabetes patients who could benefit from islet cell replacement therapy.

A second major factor limiting the accessibility of islet transplantation is the lack of sufficient cadaveric donor islets to meet the global demands for human islet cell replacement. More than 2,000 patients have received allogeneic islet transplantation globally since year 2000 (10). This is only a small fraction of the millions of individuals who have been afflicted with brittle T1D, not to mention an even larger number of patients with insulin-deficient T2D for which such a treatment is currently not an option. A recent report published by National Health Service (NHS) England stated that the waiting time for islet transplant was 631 days and the number of patients on the active islet transplant list in the UK is 29 by the end of September 2021 (40). In addition to the lack of suitable deceased donor pancreases, the quality of islet products is highly variable depending on the circumstances under which the donor organ is obtained, donor characteristics, the complex islet isolation and culturing process and the preservation conditions before transplant (41). The method of isolating islets by enzymatic digestion and mechanical separation can lead to potential damage of the endocrine cells. Majority of islet transplant recipients receive islets from multiple donors (2 to 4) as up to 60% of islet mass is lost within the first few days following islet infusion. While transplantation of larger islet mass (>11,000 IEQ/kg of recipient weight) over multiple islet infusions contributes to a larger mass of surviving β cells, this limits the number of patients that can receive the islet allografts (42). Efforts have been made across several islet isolation facilities to harmonize the donor selection criteria, manufacturing procedures, and lot release attributes, but this remains a huge undertaking to be controlled at all phases and implemented at a larger scale (43, 44).

### 2.3 Xenogeneic islets as an alternative primary cell source for transplantation

Xenogeneic islets, in particular porcine islets, have been explored as an alternative primary cell source to supplement the supply of primary human islets for transplantation. Porcine islets are more readily available and possess functional characteristics that make them a suitable substitute for human islets. They have weaker immunogenicity and porcine insulin is structurally similar to human insulin (with one amino acid difference that is alanine in pigs and threonine in humans) (45). Major hurdles need to be overcome for xenografts to be a feasible alternative in the clinic. These include physiological incompatibility and immunological reaction to non-human donor tissues that trigger both innate and adaptive barriers of the immune system, resulting in rejection. In addition, xenotransplantation presents the potential risk of zoonosis and porcine endogenous retrovirus (PERVs). As such, previous studies have evaluated the feasibility of xenotransplantation of porcine islets into non-human primates (NHPs). One study showed that an anti-CD40 (2C10R4) monoclonal antibody-based immunosuppressive regimen together with tacrolimus was effective in circumventing graft rejection and prolonging porcine islet graft survival in diabetic rhesus monkeys, with median survival (serum porcine C-peptide concentration of >0.15 ng/mL) of 60 days. All monkeys also received anti-thymocyte globulin, cobra venom factor (CVF), adalimumab, and sirolimus (46). In another study, a newly engineered anti-CD40L-specific monoclonal antibody AT-1501 was tested in a cynomolgus macaque model that had undergone intrahepatic islet allotransplantation. The study showed that AT-1501 enabled long-term graft survival with higher C-peptide levels detected compared with conventional immunosuppression (47). AT-1501 was modified to minimize risk of thromboembolic complications that were previously reported for CD40L-based therapies in clinical trials, and therefore appears to be a promising and safe agent for further testing. Another strategy to enhance graft survivability is to utilize genetically-modified pigs with alterations in expression of known xeno-antigens, and modification of the complement and coagulation systems to improve immunological compatibility between pigs and NHPs (48). In one example, cardiac xenografts from genetically-modified pigs with alpha 1-3 galactosyltransferase gene knockout, expression of human complement regulatory protein CD46 and human thrombomodulin, were transplanted into baboons (49). The pre-transplant immunomodulatory induction regimen included anti-thymocyte globulin and 2C10R4 antibody, followed by maintenance with intensively-dosed 2C10R4 antibody and mycophenolate mofetil (49). This combination of genetic modifications and immunosuppressive regimen resulted in sustained survival of the xenografts with median of 298 days up to the longest of 945 days observed (49).

In further attempts to reduce immune rejection after xenogeneic islet transplantation, porcine islets may be encapsulated in a protective layer to avoid immune cell recognition. In one study, neonatal porcine islets were encapsulated in a stable and permeable alginate gel and enclosed in a biocompatible, immunoprotective membrane, and transplanted in the abdominal cavities of immunocompetent diabetic mice. Islet xenograft survival, rapid lowering of blood glucose and long-term glycemic control for >200 days was achieved without any immunosuppressants (50). Furthermore, the devices were shown to retain their integrity after they were retrieved and re-transplanted in new immunocompetent diabetic mice. In a clinical study, alginate-based encapsulation of neonatal porcine islets were transplanted into the peritoneal cavity of eight T1D patients without immunosuppression at up to 20,000 IEQ/kg body weight over two separate transplantations (51). The procedure was shown to be safe with no PERVs infection detected. Some fibrosis of the microcapsules were observed post-transplant, however long-term efficacy was shown with HbA1c <7% over more than 600 days and significant reduction of serious unaware hypoglycemia (51). More encapsulation studies involving porcine islets are discussed in a later section and in **Table 1**, which lend support to the clinical benefit provided by porcine islet xenotransplantation in T1D patients.

**Table 1:** Summary of studies investigating the use of encapsulation (micro/macro) technologies for immune isolation of allogeneic primary islets or stem cell-derived islets for cell transplantation.

Strategy	Encapsulation Material/Device	Cell type	*In vivo* transplantation in humans or animal models (If any)	Outcome of transplanted cells	Ref
**Microencapsulation**	Chitosan hydrogel	Wistar rat islets	Yes, diabetic C57BL/6J mice	Encapsulated islets secreted insulin in response to glucose stimulation, reduced blood glucose levels for four weeks, and resulted in faster glucose disappearance rate after IPGTT compared to naked islets. Immunostaining confirmed insulin-positive cells in the graft and negative staining for T-cell lineages and monocyte/macrophages.	(52)
	Alginate/polyaminoacidic-based (patented)	Human islets	Yes, T1D patients (non-immunosuppressed)	Improved HbA1c levels with positive serum C-peptide response for 3 years post-transplant. Absence of immune infiltration observed by negative expression of anti-MHC class I-II and GAD65 antibodies 3 years post-transplant.	(53)
	Alginate/poly-L-lysine/alginate (APA)	Neonatal porcine islets	Yes, T1D patients (non-immunosuppressed), diabetic C57BL/6J mice	Reduced unaware hypoglycemia events in all patients. HbA1c < 7% achieved in 4 of 14 patients (from 1 of 14 at baseline). Reversal of diabetes and positive porcine C-peptide in mouse study.	(54)
	Alginate/poly-L-lysine/alginate (APA)	Neonatal porcine islets	Yes, T1D patients (non-immunosuppressed)	Improved HbA1c < 7% for >600 days with reduced frequency of unaware hypoglycemia events.	(51)
	Multiple alginate sphere formulations with chemically modified alginate derivatives	Cynomolgus monkey islets	Yes, non-diabetic macaques (non-immunosuppressed)	Allogeneic islets encapsulated with Z1-Y15 alginate derivative retained high viability, were glucose-responsive 4 months post-implantation in the bursa omentalis. Reduced macrophage infiltration and foreign-body reaction (FBR) and pericapsular fibrotic overgrowth (PFO) score in encapsulated islet grafts.	(55)
	Alginate polymer incorporated with immunomodulatory chemokine CXCL12	hESC-derived β cells	Yes, diabetic C57BL/6J mice	Enhanced insulin secretion of β cells, accelerated normalization of hyperglycemia with glycemic correction lasting >150 days. Limited infiltration of effector T cells, macrophages and increased recruitment of Foxp3^+^ regulatory T cells to the islet grafts.	(56)
**Macroencapsulation**	Collagen-covered device	Neonatal porcine islets combined with Sertoli cells	Yes, T1D patients (non-immunosuppressed)	Two of 4 patients had significant reduction in insulin requirement maintained up to 4 years. Porcine insulin following glucose stimulation was detectable up to 4 years. Presence of insulin-positive cells from the explanted grafts were observed in all patients post-transplant.	(57)
	Semi-permeable ethylene-vinyl alcohol copolymer membrane	Mouse pancreatic β cell line MIN6	Yes, diabetic C57BL/6 mice	Lowered blood glucose levels for 30 days in diabetic mice, no host cells within device found, no difference in circulating inflammatory cytokines in mice with and without transplant.	(58)
	TheraCyte™ device	Lewis rat islets	Yes, diabetic Wistar-Furth (WF) rats	Graft function was maintained for 6 months in both immunized and nonimmunized rats. Immunized rats showed high IFN-γ producing CD8^+^ T cells as compared to control rats transplanted with encapsulated islets.	(59)
	Sernova Cell Pouch	Syngeneic mouse islets	Yes, diabetic BALB/c mice	Restored glycemic control and showed glucose-responsiveness for 40 days. Islets within cell pouch were stained positive for insulin, glucagon, and endothelial cells.	(60)
	TheraCyte™ macroencapsulation device	Wild-type C57BL/6 neonatal pancreatic tissue	Yes, T1D RIP-LCMV.GP mice	Lowered blood glucose and the onset of diabetes was prevented in some recipients. Absence of CD8^+^ T cells in the vicinity of encapsulated C57BL/6 grafts.	(61)
	VC-01 (PEC-Encap); Physical barrier that protects transplanted grafts from host immune cell infiltration	hESC-derived pancreatic endoderm progenitor cells	Yes, T1D patients (non-immunosuppressed)	Prolonged cell survival for 24-months and positive staining for pancreatic islet cell markers, NKX6.1, insulin and glucagon was observed. No evidence of autoimmune rejection based on a panel of immune function markers.	(62)
	βAir device with two compartments: a refillable oxygen tank and an alginate and polymembrane covered chamber for immune isolation	Allogeneic human pancreatic islets	Yes, T1D patients (non-immunosuppressed)	Islet survival for 3-6 months, however, limited functionality, minute circulating C-peptide levels and no benefit on metabolic control was observed. Fibrotic tissue with immune cells were formed surrounding the capsule.	(63)
	VC-02 (PEC-Direct); non-immunoprotective to allow direct vascularization of implanted cells	hESC-derived pancreatic endoderm progenitor cells	Yes, T1D patients (immunosuppressed)	Engraftment and insulin expression were observed in 63% of subjects. Detectable C-peptide in 35% of subjects from 6 to 24 months post-implantation though with little clinical benefit. Infiltration of host myofibroblasts into devices.	(64)
	Macro device with alginate gel microcapsules enclosed in a semipermeable membrane bag with immuno-isolation	Neonatal porcine islets	Yes, diabetic C57BL/6NCr mice	Improved glycemic control for more than 200 days. Explanted devices exhibited almost no adhesion or fibrosis and showed sustained insulin secretion.	(50)
	VX-264; “channel array” macroencapsulated β cells	Allogeneic hiPSC-derived β cells	Yes, T1D patients	Ongoing clinical trial with no disclosed outcomes yet.	(65)
	Sernova Cell Pouch	Allogeneic human islets	Yes, T1D patients	Insulin independence observed for 6 to 38 months with persistent fasting and stimulated C-peptide levels. Surviving functional islets detected in Cell Pouches excised at >90 days post-transplant.	(66)

Besides the need to address the genetic and molecular discrepancies between human recipients and xeno-organs, other challenges to note include psychosocial and ethical barriers, tension from religious beliefs, concerns for animal welfare and the use of animals for research. Nonetheless, the careful use of existing or novel immunosuppressive therapies, development of genetically-modified pigs to obtain porcine islets with better immune tolerance, and use of encapsulation to provide immune protection (to be discussed in greater detail in section 3.1) make it possible for porcine islets to be considered as another safe, functional and readily available source of primary cells for T1D patients. This will help to overcome the ongoing shortage of donor human islets.

## 3 Innovations in bio-engineering and cell-based approaches for cell replacement therapy to address immunological issues

**Figure 2:**
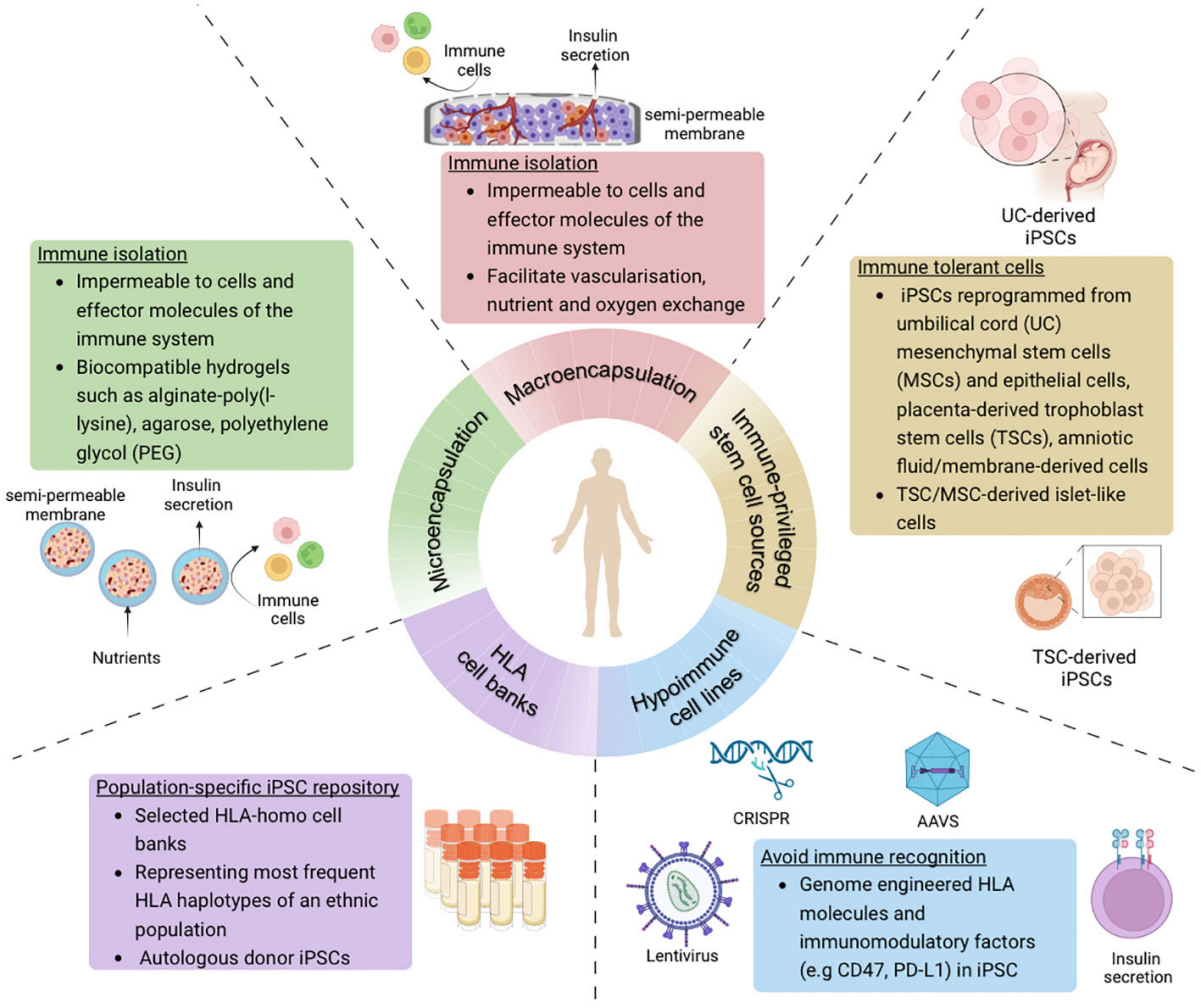
Overview of strategies to avoid immune recognition and allograft rejection of stem cell-derived islet cells in the context of cell-based therapy for diabetes treatment (Created with BioRender.com).

Various strategies have emerged to specifically address the immunogenicity of transplanted cells in islet cell replacement therapy (**Figure 2**). These not only aim to improve engraftment and functionality of the islet cells, but also make such a therapy more accessible to a wider diabetes population.

### 3.1 Development of encapsulation technologies to provide an immunoprotective environment for transplanted islets

To protect from allograft rejection and recurrence of autoimmunity, cell encapsulation is a common strategy that can provide a physical barrier to shield the islets from immune cell recognition and attack. Specifically, encapsulation helps to mediate IBMIR which is caused by islet contact with the blood and is highly detrimental to cell survivability. An ideal encapsulation device or material should be biocompatible but not biodegradable, made of a semi-permeable material to allow entry of nutrients and oxygen, and enable release of hormones and metabolic by-products into the bloodstream. Such a strategy can be based on microencapsulation or macroencapsulation (**Table 1**). Microencapsulation is a method in which islet cell clusters are individually encapsulated in spherical capsules typically 300 μm to 600 μm in diameter (67), and commonly in alginate-based hydrogels. In contrast, macroencapsulation is based on devices greater than 100 mm with capacity to house a larger mass of islet cells within the membrane. Micro-capsules have an optimal surface-to-volume ratio compared to macro-capsules that require vascularization and sufficient oxygenation to improve islet survival and function. Biomaterials used for microencapsulation are often made of natural polymers such as alginate (55), agarose (68), chitosan (52) but also synthetic polymers such as polyethylene glycol (PEG) that form a hydrogel (50, 54, 56, 69) (**Table 1**). Microencapsulation encompasses a semi-permeable membrane that has demonstrated some success in providing immune protection and mechanical stability in mice (50, 52), NHP (55) models and human studies (51, 53). However, microencapsulation requires more complex and individualized fabrication processes (70), as opposed to macroencapsulation devices that may be easier to manufacture, are more easily retrievable after implantation, and are more favourable for commercialization. Several devices that have been developed include Theracyte™ from TheraCyte Inc., βAir from BetaO_2_ Technologies, the Cell Pouch System from Sernova, and PEC-Encap (VC-01) and PEC-Direct (VC-02) from ViaCyte (now acquired by Vertex Pharmaceuticals) (**Table 1**).

The Theracyte™ planar macroencapsulation device consists of an inner hydrogel semi-permeable membrane layer for immune protection and an outer polytetrafluoroethlene membrane layer for neovascularization (71). Rodent islets encapsulated within the Theracyte™ device demonstrated survival and functionality in an immunized rat model for at least 6 months post-transplantation (59). Porcine islets transplanted subcutaneously within Theracyte™ also survived and was able to reverse diabetes up to 8 and 16 weeks in cynomolgus monkeys and NOD mice respectively (72). Another device known as the βAir bioartificial pancreas (BAP) has been tested clinically and consists of two compartments for islets, an oxygenated chamber that maintains physiological oxygen pressure, covered with a porous polytetrafluoroethylene (PTFE) membrane impregnated with alginate to provide the immunoprotective barrier (73–75). Stable graft function and insulin secretion were observed in NHP models of diabetes (73) and human patients (75) who received βAir containing porcine islets and human islets respectively, both in the absence of immunosuppressants, though complete insulin independence was not achieved. In one Phase I clinical study, βAir containing allogeneic human islets were subcutaneously implanted in T1D patients (clinicaltrials.gov: NCT02064309) (63). Although the transplanted islets survived 6 months post-transplantation, limited functionality was observed based on minute levels of circulating C-peptide with no impact on glycemic control in the patients (63). Additionally, fibrosis or inflammation were observed on the surface of the chamber. Another promising technology is the proprietary Cell Pouch system developed by Sernova, which is an implantable device that provides a vascularized tissue matrix for cells in addition to local microencapsulation of cells in polymer spheres. The Cell Pouch is undergoing testing in T1D patients in an ongoing Phase I/II clinical trial (clinicaltrials.gov: NCT03513939). In an interim update, patients with islet transplants in the 8-channel Cell Pouches were found to achieve insulin independence for as long as 3 years as a result of both functional islet grafts in the Cell Pouches supplemented by a modest intraportal islet transplant top-up through the portal vein (66). Additionally, a second version of the Cell Pouch with higher capacity is being evaluated and early patient data so far revealed persistent serum C-peptide levels detected from a single islet transplant in the 10-channel Cell Pouch (66). The company announced a collaboration with Evotec to test out human induced pluripotent stem cell (hiPSC)-derived islet cells in the Cell Pouch system in future clinical trials (66).

In another Phase I/II clinical trial by ViaCyte, human embryonic stem cell (hESC)-derived pancreatic endoderm cells (PECs) were encapsulated in a cell-impermeable device designed to be immunoprotective against recipient immune systems (clinicaltrials.gov: NCT02239354). The macroencapsulation devices containing cells (also known as VC-01 or “PEC-Encap”) were implanted subcutaneously in T1D patients in the absence of immunosuppression (62, 76), and evaluated for efficacy, tolerability, and safety. Formation of neovasculature was observed in the grafts and the PECs were able to mature *in vivo* into insulin-expressing β cells, as shown by immunohistochemical staining for pancreatic islet cell markers (NKX6.1, insulin, and glucagon) (62). VC-01 was found to be safe, well-tolerated and immunoprotective with evidence of prolonged cell survival up to 24 months. However, some inconsistency of cell survival was observed amongst subjects due to varying foreign body responses in the host (62, 76). Furthermore, no evidence of insulin secretion was found due to chronic damage to islets resulting from device fibrosis (62). This suggested that the macroencapsulation device, although well-tolerated in recipients, resulted in poor long-term engraftment and diminished efficacy due to poor oxygenation and nutrient supply to the transplanted cells. In efforts to mitigate cell loss due to device fibrosis, a subsequent version of the combination product (also known as VC-02 or “PEC-Direct”) was developed to include engineered portals in the device to enable direct capillary permeability and facilitate better vascularization to the implanted cells (clinicaltrials.gov: NCT03163511) (76). However, this was a non-immunoprotective device and patients still required immunosuppression to limit allo- and autoimmune responses. This time, patients exhibited meal-stimulated C-peptide secretion following maturation of the PECs *in vivo* and achieved the target blood glucose range for longer periods (26 weeks) as compared to VC-01 (76). Subjects in which substantial cell engraftment were observed after evaluating the explants were shown to have higher meal-responsive C-peptide levels during the follow-up period as compared to those with poor cell engraftment (64). While VC-02 is generally safe and well-tolerated, the side effects of immunosuppression accounted for majority of adverse events (AEs). Another study utilizing the same VC-02 device but with an optimized membrane perforation increased the initial implanted cell dose (14 x 10^6^ cells per kg body weight) such that it is within the range of that used for intrahepatic primary islet transplants (6-18x 10^6^ cells per kg body weight) (20, 77, 78). After 6 months post-transplantation, only 3 of 10 patients achieved C-peptide levels ≥0.1 nmol/L with reduced insulin dependence, and the detectable β cell mass in the retrieved implants was found to be less than 5% of the initial cell mass, indicating high cell loss and limited efficacy from the device-delivered PECs (78). This could be due to insufficient vascularization in the devices to support the metabolically functional β cell mass. Further optimization remains needed to increase the efficacy of the encapsulated PECs to be comparable to that of conventional human primary islet transplantation. These outcomes suggest that the macroencapsulation devices not only need to prevent entry of immune cells, but also facilitate (even encourage) vascularization to enable better cell survival and maturation into functional β cells and reduce infiltration of fibroblasts into the devices.

In another recent effort to evaluate macroencapsulated stem cell-derived islets in the absence of immunosuppressants, Vertex Pharmaceuticals, who has an ongoing first-in-human Phase I/II clinical trial (clinicaltrials.gov: NCT04786262) for their allogeneic stem cell-derived, fully differentiated islet cells (79), revealed the development of their second cell therapy program (clinicaltrials.gov: NCT05791201) investigating the islet cell product encapsulated in a “channel array” device and implanted subcutaneously (65). Though the design of the device used in the trial has not been disclosed, based on publicly-available patent filing information from the company, such a device would have a thickness of at least 300μm, an average pore size ranging from 5 nm to 2500 nm and comprising 1 x 10^6^ to 1 x 10^9^ (PCT/US2018/053665). The proprietary design also showed deformation of the membrane to a formed configuration instead of a flat configuration, with channels that enable vascularization in and around the device. The pore size had to be fine-tuned to ensure long-term structural integrity while allowing release of insulin and restricting leakage of cells out of the device (PCT/US2018/053665 and PCT/US2018/037637). It remains to be seen whether the device allows sufficient nutrient and oxygen supply, as well as provide immune tolerance in the absence of immunosuppression.

Across the numerous efforts from academic labs and commercial companies to develop and test macroencapsulation devices for islet cell transplant (**Table 1**), prevention of immune attack is found to be achievable, but a balance needs to be struck to achieve other outcomes including better vascularization and oxygenation, maximising transplanted β cell mass, preservation of β cell viability and function following implantation, and reduction of foreign body reactivity. These devices also require additional unique considerations related to manufacturing and regulatory oversight as medical devices for use in a clinical setting, evaluation of the biocompatibility of the materials, and selection of transplantation site and protocol given the larger size of the devices.

### 3.2 Combining hiPSC technology and genetic engineering to generate hypoimmune cells

Since the use of hiPSC technologies became widespread, hiPSCs have proven to be highly versatile and amenable to genetic manipulation. The generation of functional hiPSC-derived islets (SC-islets) has also made significant headway in recent years, making it possible for regenerative medicine to be part of a not-so-distant future in diabetes therapy. The journey of developing SC-islets in the lab to be as close to their primary human islet counterparts as possible, and the promise of using these cells as off-the-shelf therapy for islet cell replacement, have been extensively discussed in other recent reviews (80–82). Previously, promising preliminary clinical results had been released from Vertex Pharmaceuticals on their ongoing first-in-human Phase I/II clinical trial of lead candidate VX-880 (clinicaltrials.gov: NCT04786262), which is a hiPSC-derived, fully differentiated islet cell product administered to T1D patients in a similar fashion as primary human islets, in the presence of intensive chronic immunosuppressive therapy (83). Six patients with a history of undetected insulin secretion tolerated the therapy well, demonstrated islet cell engraftment with production of endogenous glucose-stimulated insulin and had improved glycemic control. Patients that were followed up at the 1-year mark also displayed successful elimination of SHEs and reduction in HbA1c <7.0% (83). This was a landmark shift from ViaCyte’s PEC grafts, which required cellular maturation *in vivo* into functional glucose-sensing and insulin-secreting β cells, a process that cannot be monitored and qualified before transplant.

**Figure 3:**
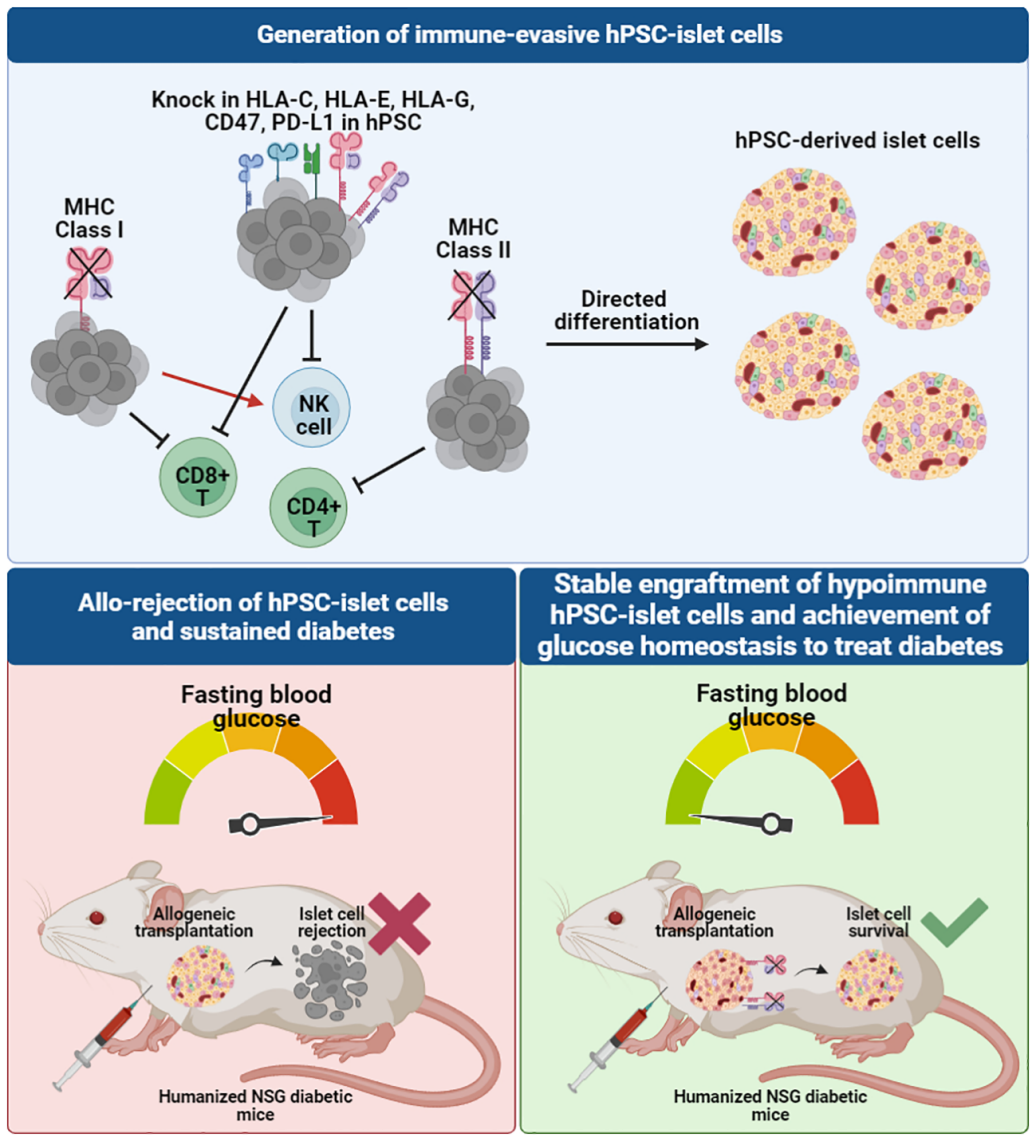
Immune-evasive hPSC-derived islet cells can be developed through genome-editing of the hiPSC source to knock out MHC class I and II molecules and knock in other immunomodulatory markers to evade different T cell and NK cell recognition, creating a tolerogenic microenvironment for allogeneic transplantation. When transplanted in humanized diabetic mouse models, unedited allogeneic hiPSC-derived islet cells face graft rejection, whereas hypoimmunogenic allogeneic hiPSC-derived islet cells survive and are able to rescue diabetes to achieve normal blood glucose levels in mice.

In combining SC-islet differentiation protocols and genome engineering techniques (84–89), several novel approaches have been employed to generate human islet cells that are protected from immune rejection, potentially eliminating, or reducing the need for systemic immune suppression and/or encapsulation. These immune evasive strategies typically work by either artificially elevating immune suppressive proteins (e.g. immune checkpoint manipulation) or removing receptors important for immune cell recognition on the cell surface (**Figure 3**). An essential component of innate and adaptive immune responses is the major histocompatibility complex (MHC) class I and II molecules which serve to present foreign antigens to the cell surface for recognition by the host immune system. In humans, these MHC molecules, also known as human leukocyte antigens (HLA), are highly polymorphic with almost 10,000 alleles. Immune rejection of hiPSC-derived cells or tissues from an allogeneic donor are mediated through these MHC molecules, limiting the survivability and therapeutic potential of the transplanted cells. Immunological mechanisms governing allograft rejection occurs in two stages: (1) non-specific innate responses predominate in the early phase, and (2) antigen-specific adaptive responses by antigen presenting cells (APCs) and dendritic cells (DCs) that result in recognition of donor antigens by host T cells (90). Both innate and adaptive immunity contribute to acute or chronic graft rejection.

To prevent innate immune rejection and further suppress adaptive immune responses, various groups have developed genetically engineered hypoimmunogenic hiPSCs or hESCs through modification of selected HLA genes and other immunomodulatory factors, and evaluated the ability of these pluripotent stem cells and their derivatives to escape immune recognition (91–93). **Table 2** provides a summary of key studies that developed hypoimmunogenic cells and evaluated the ability of the cells to evade the host immune response in both *in vitro* and *in vivo* assays.

**Table 2:** Strategies for developing and evaluating hypoimmunogenic stem cell-derived islet cells, primary islets, and other cell types.

Experimental strategy	Cell type evaluated	Outcome of *in vitro* validation	Outcome of *in vivo* validation	Reference
Pancreatic islet cells or β cells				
CRISPR/Cas9 knockout of *B2M* and *CIITA*, expression of CD47 by lentiviral transduction	miPSC and miPSC derived endothelial cells (EC), smooth muscle cells (SMC), cardiomyocytes (CM).	Enzyme-linked immunospots assay (Elispots) with splenocytes recovered 5 days after transplantation showed that WT miPSCs had strong IFN-γ and a moderate IL-4 response, as compared to engineered miPSCs that did not induce any antibody response in allogeneic mice.	*In vivo* monitoring of luciferase expression from transplanted cells showed that all three WT miPSC derivatives survived up to 50 days in syngeneic C57BL/6 mice, whereas WT miPSC derivatives were rejected in allogeneic mice, in the absence of immunosuppressants. In contrast, engineered miPSC-derived ECs, SMCs showed 100% long-term survival in both syngeneic and allogeneic mice.	(91)
	hiPSCs and hiPSC derived ECs and CMs	Mice transplanted with WT hiPSCs, and hiPSC-derived ECs and CMs demonstrated strong IFN-γ response and elevated IgM levels, as compared to recipients of engineered hiPSCs that did not mount an IFN-γ response or cellular or humoral immune response. Engineered hiPSC derivatives did not trigger NK cell activation or NK cell killing in killing assays.	WT and engineered hiPSCs were injected into allogeneic humanized NSG-SGM3 mice. *In vivo* monitoring of luciferase expression demonstrated WT hiPSC derivatives were rejected, whereas engineered hiPSC derivatives showed stable luciferase signals over time and long-term graft survival (50 days).	
Expression of PD-L1 by lentiviral transduction in hiPSCs	hiPSC-derived β-like cells	Reduced expression of immune (CD45^+^) cells in recovered grafts based on *ex vivo* flow cytometric analysis.	Kidney capsule transplantation of PD-L1-expressing β-like cells in C57BL/6J diabetic mice provided sustained control of blood glucose, as compared to those lacking PD-L1 expression. Glycemic control correlated with detectable serum human C-peptide and glucose homeostasis was observed for up to 50 days in immune-competent mice.	(88)
CRISPR/Cas9 knockout of *HLA-A/B/C* and *CIITA* while retaining HLA-A2^R^ in hESCs	hESC-derived β-like cells	Retaining expression of HLA-A2 in combination with HLA-E expression reduced NK cell activation in NK cell degranulation assays. Flow cytometric analysis of mice splenocytes and peripheral blood demonstrated absence of T cells (CD45) and NK cells (CD3) 4 weeks post-transplantation. Hence, suggesting resistance to T cell and NK cell cytotoxicity.	Transplantation in the spleen of immunodeficient NSG and NSG-MHC^null^ mice followed by luciferase monitoring and survival of grafts with HLA-A2^R^ cells up to 16 weeks post-transplantation	(87)
CRISPR/Cas9 knockout of *B2M*, overexpression of PD-L1 and HLA-E in hESCs	hESC-derived β-like cells	Measurement of luminescence demonstrated *B2M* knockout SC-islets exhibited significantly improved survival compared to WT SC-islets when IFN-γ treated SC-islets were co-cultured with PBMCs *in vitro*.	No significant difference in blood glucose levels of mice transplanted with WT and *B2M* knockout SC-islets under the kidney capsule of diabetic humanized NSG-MHC class I/II knockout mice. However, upon injection of mismatched HLA-A2 PBMCs, WT SC-islets were rejected within 2 weeks resulting in loss of *in vivo* graft function. In contrast, *B2M* knockout SC-islets showed delayed graft rejection while retaining some *in vivo* graft function as demonstrated by GSIS.	(86)
CRISPR/Cas9 knockout of *B2M* and *CIITA*, expression of CD47 by lentiviral transduction	Primary human pancreatic islets, hiPSC-derived islets	Reduced or lack of NK cell or macrophage killing of engineered hypoimmune primary pseudo-islets and iPSC-derived islets based on in vitro impedence killing assays.	Transplantation in the hindlimb muscles of immunocompetent, diabetic humanized NSG-SGM3 mice, followed by glucose monitoring. WT islet grafts were fully rejected over 7 to 10 days, whereas hypoimmune islets survived, engrafted, and achieved glycemic control for up to 29 days, as shown by *in vivo* luciferase assay.	(85)
CRISPR/Cas9 knockout of *B2M* and *CIITA*, expression of macaque CD47 by lentiviral transduction in hiPSCs	hiPSCs	Serum collected from rhesus macaque transplanted with WT islets demonstrated a peak in total IgM (after 7 days) and IgG (after 13 days) donor specific antibodies (DSA), based on antibody-dependent cellular cytotoxicity (ADCC) and macrophages or complement-dependent cytotoxicity (CDC) assays. In contrast, rhesus macaque transplanted with hypoimmune cells did not induce DSAs and did not undergo ADCC or CDC cytotoxicity.	Hypoimmune hiPSCs injected subcutaneously in the back of immunocompetent allogeneic rhesus macaque demonstrated unrestricted survival for 16 weeks, whereas WT cells were rejected within 6 weeks, as shown by *in vivo* luciferase assay.	(84, 89)
CRISPR/Cas9 knockout of *B2M* and *CIITA*, expression of macaque CD47 by lentiviral transduction in hiPSCs	hiPSC-derived β-like cells	Histology staining demonstrated the injected sites for WT islets had no evidence of injected cells after 28 days, whereas sites injected with hypoimmune β-like cells had well-formed islets with no apparent inflammation observed.	WT and hypoimmune β-like cells were injected into the thigh muscle of immunocompetent allogeneic humanized NSG-SGM3 mice. Reduction in fasting hyperglycemia, and ameliorated diabetes in mice injected with hypoimmune β-like cells were observed up to 28 days. In contrast, WT islet transplants showed no effect on glucose levels in diabetic mice.	(84)
CRISPR/Cas9 knockout of *B2M* and *CIITA* and expression of rhesus macaque CD47 by lentiviral transduction	Primary rhesus macaque islets	Flow cytometric analysis of WT and hypoimmune rhesis macaque demonstrated nulled expression of HLA Class I, no difference in HLA Class I expression and significant increase in CD47 expression as compared to WT islets.	WT and hypoimmune islets were injected into the quadricep muscle of immunocompetent rhesus macaques. Hypoimmune islets achieved long-term survival up to 40 weeks, whereas WT islets were rejected within 1 week, as demonstrated by *in vivo* luciferase assay.	(84)
Other cells				
AAV-mediated knockout of *HLA-A/B/C* and knock in of *HLA-E* in hESCs	hESC-derived CD45+ hematopoietic derivatives	T cell-mediated cytotoxicity assay demonstrated that CD8^+^ T cells efficiently lysed B2M^+/+^ CD45^+^ cells, but did not kill B2M^-/Edimer^, and B2M^-/Etrimer^ cells *in vitro*.	Luciferase-expressing B2M^+/+^ and B2M^-/Etrimer^ ESC-derived teratomas and primed allogeneic CD8^+^ T cells were subcutaneously injected in immunodeficient NSG-B2M knockout mice. More growth was observed in B2M^-/Etrimer^ teratoma as compared to B2M^+/+^ after CD8^+^ cell infusion.	(92)
CRISPR/Cas9 knockout of *HLA-A/B/C* and *CIITA*, knock in of PD-L1, *HLA-G* and *CD47* in AAVS1 site in hESCs	hESC-derived endothelial cells (ECs) and vascular smooth muscle cells (VSMCs)	WT and engineered hESCs-derived ECs were pretreated with IFN-γ and co-cultured with carboxyfluorescein succinimidyl ester (CFSE)-labeled allogeneic CD3^+^ T cells. Flow cytometric analysis demonstrated reduced proliferating T cells (CD3^+^), reduced activation markers (CD69^+^ and CD154^+^) in engineered ECs as compared to WT. Similarly, allogeneic NK cells co-incubated with engineered hESC-VSMCs demonstrated significantly reduced NK cell degranulation compared to WT, as shown by flow cytometric analysis of CD107a.	WT and engineered hPSCs were subcutaneously injected in immunodeficient mice and monitored for teratoma formation over the course of 4 to 6 weeks. WT teratomas displayed a slower increase in volume compared to engineered teratomas. Furthermore, histology staining and qPCR analysis of human effector T cell markers CD8 and IL-2 had demonstrated reduced T cell infiltration in engineered cell lines compared to WT.	(93)
CRISPR/Cas9 knockout of *HLA-A/B* (haploid *HLA-C*) or *B2M* knockout in hiPSCs	hiPSC-derived CD43^+^ blood cells and cardiomyocytes	^51^Cr release assays performed with HLA-reactive T cells demonstrated that *HLA-A/B* knockout (haploid *HLA-C*) and *B2M* knockout hiPSC-CD43^+^ blood cells could evade CD8^+^ T cell-mediated cytolytic activity, but not in WT cells. Flow cytometric analysis measuring CD107a expression in NK cells co-cultured with hiPSC-CD43^+^ blood cells, had demonstrated significantly lower NK cell-mediated cytotoxicity in engineered hiPSC-CD43^+^ blood cells compared to WT.	Luciferase-expressing iPSC-CD43^+^ blood cells were pre-treated with IFN-γ and injected intraperitoneally into NRG mice. After transplantation of CD43^+^ blood cells, CD8^+^ T cells were injected. *In vivo* luciferase monitoring demonstrated significantly higher survival ratio of HLA-A/B knockout (haploid HLA-C) CD43^+^ blood cells as compared to WT after 7 days. *HLA-A/B* knockout (haploid *HLA-C*) also showed significantly better survival *in vivo* when NK cells were injected after transplantation of hiPSC-CD43^+^ blood cells, as shown by *in vivo* luciferase expression.	(94)

The potential application of hypoimmunogenic hPSC-derived islet cells for cell replacement therapy has been demonstrated in several pre**-**clinical studies in immunocompetent diabetic animal models (85–89). Simultaneous deletion of beta-2-microglobulin (B2M), a component of MHC class I-encoded HLA-A/B/C molecules, and MHC class II transactivator CIITA in hiPSCs (93) and primary human islets (85) resulted in ablation of cytotoxic CD8^+^ and CD4^+^ helper T cell responses. When evaluated for immune tolerance *in vitro*, B2M and CIITA knockout hESC- and hiPSC-derivatives were co-cultured with T cells, NK cells and PBMCs and were found to be resistant to T cell, NK cell, and complement-dependent cytotoxicity and macrophage engulfment (86–88, 93, 94) (**Figure 3**). The expression of the non-classical MHC molecules, HLA-E and HLA-G, were also found to contribute to establishing an immunosuppressive microenvironment by binding the inhibitory NK cell receptors CD94/NKG2A and facilitating the escape of human tumors from the host immune response (95). A previous study showed that HLA-A/B/C knockout hESCs and their differentiated CD45+ cells and RPE cells that overexpress HLA-E are resistant to CD8+ T cell cytotoxicity and NK cell-mediated lysis in both *in vitro* and *in vivo* models (92).

Another immunomodulatory effector is CD47 which acts as an anti-phagocytic ligand to inhibit activation of the innate immune system (96). CRISPR/Cas9-mediated knockout of B2M and CIITA together with lentiviral transduction-based overexpression of CD47 in mouse and human iPSCs were effective in generating hypoimmunogenic derivatives that did not trigger NK cell activation *in vitro* (91). Loss of immunogenicity was also recapitulated *in vivo*, as observed by significant improvement in graft survival post-injection of mouse iPSC- and hiPSC-derived smooth muscle cells (SMCs), endothelial cells (ECs) and cardiomyocytes (CMs) into the right thigh muscle of immunocompetent C57BL/6 mice (91). This approach was replicated using hiPSC-derived SMCs, ECs and CMs that were transplanted into humanized NSG-SGM3 mice. Humanized mice have been widely used as a pre-clinical *in vivo* model that recapitulates the human context, in this case the human immune system. NSG-SGM3 mice used in the study supports the stable engraftment of human myeloid lineages, regulatory T cell populations and production of hIL-15, thereby promoting the development and/or function of human NK cells. B2M^-/-^/*CIITA* ^-/-^/CD47^+^ hiPSC-derivatives showed sustained graft survival for more than 50 days, whereas unedited WT derivatives were rejected within 14 days (91).

A similar strategy was also validated in hypoimmune B2M^-/-^/*CIITA* ^-/-^ and CD47-overexpressing primary human islets (85). WT and hypoimmune human islets injected intramuscularly in humanized immunocompetent mice were monitored using bioluminescence imaging. WT islets were fully rejected within 7 to 10 days, exerted no beneficial effect on glucose homeostasis and no detectable C-peptide secretion after 29 days. In contrast, mice injected with hypoimmune islets showed allograft survival and achieved glycemic control, indicating that the function of allogeneic hypoimmune islets was sustained and confirming the ability of B2M^-/-^/*CIITA* ^-/-^/CD47^+^ to modulate immunogenicity and escape immune attack (85). More recently, Hu et al. also reported the successful rescue of an immunocompetent, diabetic cynomolgus monkey with allogeneic, hypoimmune B2M^-/-^/*CIITA* ^-/-^ and CD47-overexpressing primary rhesus macaque islets (89). C-peptide remained detectable in the monkey serum and insulin independence was achieved without immunosuppression for up to 6 months (89). These results show that hypoimmune islets can be protected from immune rejection while maintaining graft function *in vivo*.

Programmed death-ligand (PD-L1) is an immune checkpoint protein that has also been in the spotlight as it plays a role in suppression of adaptive immune response by inducing a co-inhibitory signal in activated T cells and promoting T cell apoptosis (97). Gerace et al. reported genetically engineered B2M-deficient hESCs with PD-L1 overexpression in addition to HLA-E overexpression. The authors found that in response to PBMC injection, WT SC-islets transplanted under the kidney capsule of diabetic humanized NSG-double knockout (hu-NSG-DKO) mice were destroyed within 2 weeks due to PBMC-mediated cytotoxicity, whereas graft rejection was delayed when B2M^-/-^ SC-islets were transplanted. At 7 weeks post-PBMC injection, B2M^-/-^ SC-islets gave rise to positive glucose-stimulated insulin secretion (GSIS) outcomes and were able to reverse diabetes in mice whereas graft function was lost in mice transplanted with WT SC-islets (86). These results suggested that removal of B2M could delay the rejection of the SC-islets, though it is possible that the grafts may eventually be completely rejected in longer term studies. The authors however showed that overexpression of PD-L1 in the B2M^-/-^ SC-islets did not protect the cells from xeno-rejection, and that overexpression of HLA-E did not provide additional protective benefit against NK cell cytotoxicity in their model. Instead, they found that SC-islets engineered to secrete tolerogenic cytokines such as IL-10 and TGF-β are protected against xeno-rejection likely due to recruitment of Tregs to induce a tolerogenic environment. The engineered SC-islets could reverse diabetes in NOD mice up to 8 weeks post-transplantation. Another group however showed that PD-L1 overexpression could create an immune-evasive microenvironment for SC-islets transplanted in immunocompetent diabetic mice, by restricting T cell activation and delaying graft rejection (88). While both SC-islets with and without PD-L1 overexpression had similar efficacy in restoring glycemic control in diabetic mice within a few days, the functionality of the islets lacking PD-L1 was quickly lost. On the other hand, islet cells overexpressing PD-L1 provided sustained blood glucose homeostasis, with human C-peptide levels correlating with glycemic control for more than 50 days (88).

While most of the reported work on the development of hypoimmune cells have been within pre-clinical settings, new efforts are now emerging to evaluate the cells in clinical studies. CRISPR Therapeutics (previously in conjunction with ViaCyte) is conducting first-in-human Phase I clinical trials with an investigational, allogeneic, gene-edited, hypoimmune stem cell-derived PECs for T1D (clinicaltrials.gov: NCT05210530, NCT05565248). The cells are also encapsulated in a device to be implanted in patients without immunosuppressive therapy. Vertex Pharmaceuticals also announced that it will license CRISPR Therapeutics’ gene-editing technology to add value to their ongoing efforts in the clinical development of iPSC-derived islet cell therapy for T1D (98). Although details of the partnership were not disclosed, the collaboration is likely to explore the development of hypoimmune, fully-differentiated iPSC-derived islet cells for transplantation into T1D patients without immunosuppression (with or without encapsulation). These studies aim to establish whether generation of universal hypoimmunogenic hPSCs differentiated into insulin-producing islets could provide long-term survival due to evasion of immune-mediated detection and killing. Positive outcomes from the trials will mean maximising the efficacy of the transplanted islet cell mass and providing a longer term, immunosuppression-free curative therapy for allogeneic recipients.

Despite the attractiveness of genome-edited hypoimmune cells as a cell source for allogeneic cell therapy, the long-term safety and efficacy remains to be ascertained as most studies are currently conducted *in vitro* or in animal models. As hypoimmune cells can escape immune detection, this raises concerns on cell malignancy, especially for hPSC derivatives which may give rise to tumour formation in the presence of any residual hPSCs or incompletely differentiated cells in the graft. Furthermore, CRISPR-based genome editing may induce unintended off-target genomic mutations that may contribute to aberrant gene expression that may contribute to malignancy (99–101). Therefore, tumorigenicity tests as well as evaluation of the genomic and epigenomic stability of modified cell lines remain essential to qualify any hPSC-based cell product for clinical applications. New generations of gene-editing tools have also been developed to improve on the design of nucleases, repair templates and analysis of potential off-target editing to reduce tumorigenicity risk and unintended outcomes (102–104). For instance, expression systems containing suicide gene constructs can potentially eliminate any tumorigenic cells that arise, to safeguard against tumour formation in hPSC-based cell therapies (105). A recent study showed that a combination of immune-cloaked mouse ESCs in which several immunomodulatory transgenes are being expressed, coupled with a genomically integrated FailSafe suicide transgene system, was able to generate various ESC-derived tissues that possess immune privilege. Allogeneic cells transplanted in these ‘artificially-created’, immune-privileged sites could be protected from rejection for months (106). The FailSafe system is a patented technology which creates a transcriptional link between the suicide herpes simplex virus thymidine kinase gene (*HSV-TK*) and a cell division gene (*CDK1*) to enable killing of any undesired dividing cells using a pro-drug treatment (106–108). Such safeguards help to improve the safety profile of cell therapy products, particularly those that are engineered to be more immune tolerant. For similar safety reasons, having the cells implanted within an encapsulation device also facilitates easy removal of the cells in case there is a need for the graft to be excised.

### 3.3 Use of immune privileged stem cell sources to generate islet cells

Another strategy for transplanted grafts to be potentially shielded from the immune system involves the use of naturally immune privileged stem cell sources. Several tissues in the body are evolutionarily adapted to be protected from inflammatory immune responses, including extra-embryonic tissues such as the amnion, placenta and umbilical cord. These tissues possess immune privileges so that maternal tolerance toward fetal cells may be maintained. Specifically, these tissues contain stem cells such as umbilical cord lining mesenchymal stromal cells (CL-MSCs), amniotic MSCs and placenta-derived trophoblast stem cells (TSCs). These cells also represent valuable cell sources for the generation of hiPSCs. It is postulated that hiPSCs derived from immune privileged cells may retain some of the same genetic signatures and epigenetic memory. Whether the differentiated cells from these hiPSCs also maintain their ‘privileged’ status however remains to be tested in a cell type-specific manner. Accumulating evidence suggests that the immunogenicity of hiPSC-derived cells are cell type-dependent, as different cell types exhibit different immunomodulatory mechanisms. Retaining at least some extent of the immune privileges of the original tissue stem cells may help in resisting immune destruction in the event of allogeneic transplantation.

MSCs are multipotent stem cells with high proliferative capacity, low immunogenicity and immune modulation properties due to the expression of tolerogenic factors. Successful reprogramming of placental amniotic membrane MSCs and amniotic fluid stem cells at high efficiency has previously been shown (109–111). The hiPSCs retained the immunomodulatory signatures of the MSCs, such as absence of expression of MHC class I and II proteins, and expression of HLA-G and CD59 (109). Umbilical cord lining epithelial cells (CL-ECs) are another population of cells that do not express MHC class II molecules and co-stimulatory molecules, and express non-classical HLA-E and -G, that function to suppress maternal T cell and NK cell responses (112). Therefore they not only have low immunogenicity but also possess some immunosuppressive capacity (113, 114). In a recent study, CL-ECs differentiated into retinal pigment epithelial (RPE) cells and transplanted into mice and monkey models were found to elicit reduced pro-inflammatory responses and immune cell infiltration compared to transplanted RPE cells differentiated from skin-derived hiPSCs (115). There have been few published reports, if any, exploring differences in immune tolerance of islet cells derived from hiPSCs reprogrammed from different cell sources, representing a gap that warrants further study.

Besides using hiPSCs as the starting source of cells for differentiation into insulin-secreting islet cells, MSCs have also been directly differentiated into islet-like cells by genetic manipulation (116) or step-wise induction using specific medium and small molecules *in vitro* (117–125). Umbilical cord-derived MSCs (UC-MSCs) are attractive as a starting material as they can be obtained through pain-free and non-invasive methods, are available in abundance, and have high proliferation and differentiation capacity (118). Previous studies demonstrated that UC-MSCs do not induce allogeneic PBMC immune responses and can suppress the function of mature dendritic cells *in vitro* (113). UC-MSC-derived islet-like cell clusters also retained their immune privileged properties *in vivo* and were capable of regulating glucose homeostasis (118). Primitive stromal cells isolated from the umbilical cord Wharton’s jelly have also been differentiated directly into insulin-secreting islet-like cell clusters that express beta cell markers C-peptide and PDX1, and higher levels of secreted insulin compared to bone marrow-derived MSCs (126). Such an approach is however less widely adopted than hiPSC-based differentiation into islet cells, in part due to the lack of reproducibility in the functionality of the β-like cells derived using these methods, and the fact that MSCs experience replicative senescence, which will limit the ability to continuously generate differentiated cells at larger scale. Another immune privileged cell type of note are TSCs, which are a unique population of stem cells derived from the placenta that are fetal in origin, and that form the interface between the fetus and mother throughout pregnancy (127). They are immune tolerant as they have little to no expression of the classical MHC molecules and may also be differentiated into the different germ layers. Therefore, TSCs are another understudied cell source for allogeneic cell therapy, and attempts to derive islet cells directly from TSCs have yet to be reported.

In conclusion, the use of immune privileged stem cells as alternative cell sources for hiPSC generation or for direct differentiation into islet cells could be another strategy to eliminate or reduce the intensity of immunosuppressive therapy without the need for genome editing. Nonetheless, whether the differentiated cells retain their immune privilege, and to what extent, would be crucial to ascertain in a cell type-specific manner.

### 3.4 Comprehensive stem cell banks to facilitate HLA donor matching

It is known that the predominant mediator of allograft rejection is HLA mismatch triggering T cell-mediated rejection. HLA molecules found on the surface of most cells have an important role in enabling the immune system to recognise “self” versus “non-self” antigens (128). The MHC system in humans consists of the classical MHC class Ia (HLA-A, -B, -C), non-classical MHC class Ib (such as HLA-E, -F, -G) and MHC class II (HLA-DR, -DQ, -DM, -DP) molecules that are involved in antigen presentation to CD8^+^ T cells (129), natural killer cells (NK cells) (130), and CD4^+^ T cells (131). HLA matching is not currently a criterion for primary human islet transplantation, however retrospective studies have showed that matching at selected loci, particularly HLA-DR and HLA-B, could improve long term islet allograft survival (132, 133), which would further lead to more prolonged insulin independence in patients. Another study involving follow-up of pancreas transplants purported that the number of HLA-DR and HLA-B matches correlated with a reduction in acute graft rejection, though there was no evidence to suggest a similar correlation with graft or patient survival rate (134). In other studies featuring other tissue transplantations, HLA matching has been shown to result in reduced allogeneic immunogenicity, increased graft survival, and therefore potential reduction in the intensity of immunosuppression required (135–138). Although HLA typing to match donor and recipient antigens at selected loci is clinically feasible, incorporating HLA haplotype matching in primary islet transplantation remains challenging due to existing pressure from limited cadaveric donors and the need for islet infusions from multiple donors. Thus, an avenue that may be explored relates to the formation of a repository of hiPSCs carrying different HLA haplotypes that may be matched to many recipients within a given population. Such a repository would be made up of clinical-grade HLA homozygous hiPSCs derived from carefully selected donors with homozygous HLA types to enable HLA matching when the need for an allogeneic transplantation arises (139). Based on prior experiences from cord blood and kidney grafting studies, HLA-A, -B, and -DR have been indicated as the most important HLA loci to match for long-term graft survival, with or without immunosuppressive drugs (140–142).

Several groups across different countries have embarked on efforts to derive repositories of HLA-homozygous hiPSCs that capture the high frequency HLA haplotype backgrounds (most typically for HLA-A, -B and -DRB1) in their population. The Center for iPS Cell research and Application (CiRA) of Kyoto University runs an iPSC Stock Project that aims to establish an HLA-homozygous iPSC haplobank for most of the Japanese population (143, 144). They recently reported a clinical-grade iPSC haplobank consisting of 27 iPSC lines from seven HLA-homozygous donors that could cover 40% of the Japanese population (145). Generation of HLA-homozygous iPSC lines for coverage of other geographical populations and ethnic groups have also been shown in Korea, where ten of the most frequent HLA-homozygous lines can match 41% of the population (146, 147), in United Kingdom (139), Spain (148), and China (149). The feasibility of such an endeavour has also been explored in Brazil, where it is estimated that 3.8 million people have to be screened to obtain 559 triple HLA-homozygous cell lines covering 95% of the population (150). In Finland (151), the top ten most frequent haplotypes homozygous for HLA-A to -DQB1 were compatible with 49.5% of the population. In Australia (152), haplotyping frequencies could be estimated from existing national blood banks or cord blood banks. A probabilistic model developed by Gourraud et al. evaluated multiple ancestry backgrounds and estimated that construction of a hiPSC bank representing 20 of the most frequent HLA haplotypes in each of the European American and African American populations would require screening of 26,000 and 110,000 donors respectively (153). This would match with over 50% of the European American and 22% of the African American populations respectively (153). Population-specific hiPSC haplobanks may also be deployed for other populations, especially closely related ones compared to ethnically-diverse populations. For such a biobank to be used for clinical applications, the cell banks have to be manufactured in compliance with Good Manufacturing Practice (GMP) and be qualified as a clinical grade cell bank, which is is highly costly, resource-intensive and not a trivial undertaking. The efficiency of such an effort depends on successfully identifying the desired HLA-homozygous haplotypes in an opportunistic manner within a screened population (potentially a prohibitively large one), unless a population-wide genomic data or blood bank typing data exists that allows for donor recall by genotype. In addition, data have shown that HLA-matching alone may be insufficient for successful allogeneic engraftment, and immunosuppression may still be required (137, 154). Furthermore, tolerability of HLA-matched hiPSCs may be dependent on other non-MHC factors such as the method of reprogramming, the cell type being transplanted (different cell types have different levels of immunogenicity), and the site of transplantation (whether the site is immunologically privileged) (155).

In addition to universal and ‘super donor’ hiPSC banks that we have discussed so far, personalized donor-specific hiPSC banks can also be generated for autologous use. As protocols to reprogramme easily accessible somatic cells (such as blood) are now standardized, it is possible to generate hiPSCs successfully for many individuals, at scale. There are increasingly more solutions being developed for large scale, high throughput generation of hiPSCs (156–159). These support the potential for autologous transplantation using patients’ own iPSCs as starting material to reduce immune-mediated graft rejection and therefore eliminate or reduce the need for immunosuppressive therapy. Guha et al. demonstrated that syngeneic mouse iPSCs differentiated into the three embryonic germ layers had little to no immunogenicity when transplanted into the subcapsular renal space of preclinical models (160). Morizane et al. also demonstrated that autologous transplantation of iPSC-derived dopaminergic neurons in the brains of NHPs elicited minimal immune response (161). These studies evaluated immunogenicity in different transplant sites, and transplantation in immune-privileged sites such as the central nervous system, brain and eyes do not generally trigger an immune response as compared to other sites, hence the site of transplantation must be considered even in an autologous setting. There are also several other considerations unique to hiPSC-derived cell therapy. The immunogenicity of autologous grafts remains to be validated as potential causes of immunogenicity include immaturity of the hiPSC derivatives, the reprogramming process and extended period of culturing and passaging of hiPSCs leading to genetic and epigenetic changes, and other off-target effects when gene correction is done on the donor cell line (162). To support the notion that stem cell derivatives exhibit variable immunogenic properties, Zhao et al. showed that autologous iPSC-derived SMCs are highly immunogenic to the immune system due to the dysregulated expression of immunogenic proteins, whereas iPSC-derived RPE cells are not immunogenic (163). Therefore, hiPSC-derived cells may not retain their immune privileged properties upon differentiation, resulting in immune attack, possibly due to differing levels of expression and activity of immunomodulatory proteins during the cellular differentiation process (163). Long-term (>4 months) evaluation of graft function and immune responses need to be considered when translating pre-clinical findings to the human context. The creation of donor cell banks for patients in need would be costly and time-consuming, given that reprogramming and qualification of the cell bank, followed by subsequent differentiation into islet cells, could easily take at least a few months. This is in contrast with other currently approved autologous therapies such as chimeric antigen receptor (CAR)-T cell immunotherapy which takes 2 to 3 weeks from apheresis to cell infusion. Nonetheless, autologous hiPSC-based cell therapy remains a useful platform for evaluating the safety and efficacy of regenerative medicine treatments for disease without many of the concerns that allogenic transplantations pose (164).

## 4 Discussion

Human islet transplantation has demonstrated substantial success in improving the lives of T1D patients who were suffering from SHEs, and restoring their insulin independence. There lies a lot more potential for T1D patients and even selected T2D patients,to benefit from an islet cell replacement therapy. However, here we have discussed major obstacles that need to be overcome including the need for chronic immunosuppression, lack of sufficient organ donors, and immune responses that negatively impact on graft function.

Reduction or removal of immunosuppression is the key to being able to treat diabetes sustainably with a curative therapy, whether through organ/tissue transplant or a regenerative medicine approach. The rise of hPSC-derived islet cells for therapeutic use represents a new paradigm shift in regenerative medicine. Various strategies have been highlighted here, namely: (1) fine-tuning of the immunosuppressive regimen to reduce side effects, (2) exploring alternative primary cell sources such as porcine islets, (3) using immunoprotective encapsulation materials or devices to preserve the long-term function of the transplanted cells, (4) using hiPSC-derived islet cells and genetic engineering approaches to provide a renewable and well-characterized source of cells that can evade the host immune system, (5) harnessing the immune-privileged properties of tissue-derived stem cells to make hiPSCs or perform direct differentiation to islet cells, and (6) manufacturing a repository of HLA-homozygous hiPSCs suitable for clinical applications. In reality, it is likely that a combination of a few of these strategies will be needed. Current and future pre-clinical and clinical work will need to be at the intersection of multiple strategies, such as the use of encapsulated islet cells derived from HLA-selected hiPSC lines that have been genetically engineered to possess more immune-tolerant and safety features. However, adopting multiple strategies will also mean needing to address the shortcomings of each, and adding layers of complexity to eventual clinical translation (**Table 3**). It is likely that there is no one-size-fits-all strategy.

**Table 3:** Comparative analysis of immune evasion strategies for islet cell therapy.

	Microencapsulation Material	Macroencapsulation Device	Immune privileged stem cell sources	Hypoimmune hiPSCs	HLA-selected hiPSC repository
**Advantages**	Prevents immune cells from recognizing the transplanted cells	Prevents immune cells from recognizing the transplanted cells	Naturally possess low immunogenicity and even immunosuppressive capacity	Genetically engineered in a customizable manner to evade the host immune system	Facilitates HLA matching with large numbers of recipients
	Larger surface-to-volume ratio enables more efficient diffusion of oxygen and nutrients	Longer *in vivo* durability/stability compared to microencapsulation materials	Can be used to derive iPSCs or other cell types that may retain their immune privilege	Potentially a universal stem cell line	Provides a country/population-specific national cell resource
	Facilitates vascularization *in vivo*	Cell chambers may be refillable without device retrieval	Potentially applied without the need for immunosuppressant drugs		Potentially used with reduced intensity of immunosuppressant regimen
**Potential for clinical applications**	Can be optimized for different cell types	Can be optimized for different cell types but restricted to limited transplantation sites	Multipotent stem cells may be differentiated into a few (but limited) cell types; iPSCs may be differentiated into many different cell types	iPSCs may be differentiated into many different cell types	
	Provides flexibility for transplantation at different sites	Suitable for less invasive transplantation methods or sites (e.g subcutaneous implantation)	May be transplanted at different sites to suit different regenerative medicine applications	
**Manufacturability**	Requires manufacturing in conjunction with cells	May be mass manufactured independently of cells initially	Stem cells may be scaled up easily but have limited proliferative lifetime	Unlimited quantities of iPSCs may be generated to obtain universal cell bank	Unlimited quantities of iPSCs may be generated to obtain HLA type-specific cell bank
	Many medically-approved, biocompatible biomaterials available	Manufacturing of different layers or components required (such as inner membrane for immune protection, outer membrane for neovascularization) but may be highly tunable	High cost and resource-intensive for manufacturing clinical grade hiPSCs at scale, though with new technological developments the costs are likely to decrease in future		
**Safety**	Biocompatible materials available	Biocompatible materials available	Presence of partially differentiated cells or residual hiPSCs may pose tumorigenic risk		
	Difficult to retrieve depending on implantation site	Easy to retrieve in case of therapeutic failure or safety concerns	Difficult to retrieve depending on implantation site, especially without accompanying macroencapsulation device		
**Other limitations**	May be susceptible to enzymatic or hydrolytic breakdown in the body	Smaller surface-to-volume ratio may result in inefficient diffusion of oxygen and nutrients into and within the device	Immune privilege properties may be lost upon reprogramming and/or differentiation	Potential unintended off-target mutations from genome editing procedure	Generation of cell bank requires extensive screening and selection of donors
	Non-refillable and non-reusable	Need for vascularization to improve graft survival	Multipotent stem cells may not generate mature cell types that fully recapitulate the native function	Potential for aberrant malignant cells to escape immune detection	Large number of cell lines needed to cover majority of population
	Weak mechanical strength	Limited device volume requiring use of multiple separate devices	Immunogenicity of stem cell/hiPSC derivatives is cell-type dependent and every cell type generated needs to be evaluated		
		Limited options for transplantation site			

Immune isolation or encapsulation of islets relies on a physical barrier to protect graft function. There are many gold standard biomaterials used for encapsulation of islets (refer to **Table 1**) that are straightforward to mass produce. The long-term durability of the biomaterials *in vivo* will need to be tested and optimized in an application specific manner. For translational purposes, production of the encapsulation materials/devices need to conform with good manufacturing practices and ISO standards normally under the regulation of medical devices. Encapsulation has been tested on all of primary human islets, porcine islets and SC-islets (**Table 1**), and it is feasible for such platform technologies to be developed to suit different cell types and disease applications. Macroencapsulation devices have been shown to be applied to cardiovascular diseases (165–167) and CAR-T cell therapy (168, 169) and shown promising preclinical outcomes as well.

Although more hiPSC-based cell therapeutic products are being tested in the clinic now, indicating that safety testing can meet the regulatory barrier for clinical trial authorizations of specific products, there remains many reservations about product safety that have to be managed for each unique cell type. The creation of universal hiPSC lines that elude immune recognition can offer tremendous promise for regenerative medicine applications, beyond cell therapy for diabetes. Genetic modifications to engineer hypoimmune iPSC-derived endothelial cells and cardiomyocytes have demonstrated efficacy in treating cardiovascular and pulmonary diseases in immunocompetent allogeneic mice (170). However, these hypoimmune cells also present a safety risk after human application and need to be carefully monitored. This is due to the potential for undesirable tumorigenicity arising from residual undifferentiated, pluripotent cells in the final product. Therefore, in the presence of conventional immunosuppressive therapy, or in the case of modified hiPSCs that can escape immune surveillance, the body may not be able to detect and respond to potential malignancies. Additional genetically engineered safeguards for hiPSC-based products are being developed for elimination of aberrant cell growth (106, 171), but it is uncertain how these cells would behave in human patients over time. As for ethnic-specific HLA haplotype cell banks, though established from homozygous HLA haplotypes, they may not provide a complete match and therefore a combination of encapsulation technologies to provide additional immune isolation, and/or some use of immunosuppressants or anti-inflammatory drugs are still needed to prevent graft rejection. There are also other factors not related to MHC compatibility that can trigger immune responses, such as undesirable gene disruptions arising from the iPSC reprogramming or gene editing process, components of the culture media, and minor histocompatibility antigens (due to recognition of mutated proteins recognized as foreign antigens) even in the case of HLA-matched transplants.

As SC-islets are expected to be regulated as biologics, similar to the route that the US FDA had taken for donor-derived isolated pancreatic islets, drug manufacturing principles will apply for the regulation of the cell therapy (26). Unlike the limitations for freshly harvested and isolated primary human islets, it is possible for the sterility and potency of SC-islets, among other critical quality attributes, to be verified prior to clinical use (172). Lessons from clinical failures due to MSC product inconsistencies highlighted the need to establish appropriate product quality controls, owing to the variability in cell initiation and differentiation procedures, culture conditions and expansion processes among others (173).

There remain various ongoing efforts for improving the outcomes of islet transplantation. One area of research is on graft vascularization. Previous studies sought to improve graft re-vascularization through various methods such as transfection of tissues with mRNA encoding angiogenic growth factors (e.g vascular endothelial growth factor (VEGF-A)) (174), co-transplantation with vascular fragments (175), and pre-vascularization of the engrafted site (176, 177). Alternatively, re-vascularization of islets was also shown to be improved by resizing the islets into smaller clusters (≈150μm diameter) combined with a biocompatible polycation coating, that resulted in achievement of long-term euglycemia in immunocompetent mice up to 6 months (178). Alternative transplant sites have also been explored. For example, the intramuscular (179), gastric submucosa (180), eye (181), and perihepatic surface (182) are being investigated as alternative engraftment locations that may enhance the viability of grafts. Some immunologically privileged transplant sites enable allografts to survive for extended or even indefinite periods, however not all sites are suitable for islet transplantation in human patients due to site accessibility and potential side effects (more extensive review of alternative transplantation sites are out of the scope of this review).

Another innovative strategy to circumvent immunosuppression include co-transplantation of islets with immunosuppressive cells such as MSCs engineered to express PD-L1 and CTLA-4 (183), which act as accessory cells to induce local immunomodulation. Another study had showed that recipient-derived MSCs co-transplanted with islet allografts and MSCs infused in diabetic cynomolgus monkeys (fully MHC mismatched) after islet transplantation exhibited delayed rejection due to downregulation of memory T cells, reduced anti-donor T cell proliferation and increased Tregs (184). While promising, administration of immunosuppressive drugs and anti-inflammatory drugs albeit at reduced doses is still required, and the sustenance of the immunomodulatory effects exerted by the MSCs in the long run remains to be determined. As allogeneic MSCs provided poorer outcomes than autologous MSCs when used alone in the same study (184), the need to collect and process autologous MSCs will add to the complexity during clinical translation. Additionally, myeloid-derived suppressor cells (MDSCs), a cell population of myeloid origin that can mediate allogeneic immune responses, may potentially be co-transplanted with islet allografts to help prolong graft survival (185). Alternatively, a recent report combined cell and gene therapy by co-transplanting allogeneic islets with streptavidin-FasL-presenting microgels in the omental pouch of diabetic non-human primates (186). Using FasL as an immunomodulatory agent induced local tolerance in the absence of immunosuppression, due to increased number of FoxP3+ cells in the graft site.

Other areas that need to be addressed include reducing cost of manufacturing of the hPSC-derived islet cells, through automation, cryopreservation and better economies of scale when produced in large scale batches. Even if the risk from immunosuppression can be eliminated, there is also the question of where cell therapy falls within the pipeline of standard of care treatments for poorly controlled diabetes, in the face of insulin therapy or insulin pumps which are less invasive. This would also depend on the availability of resources to administer the cell product in the clinic, willingness to attend frequent follow-ups, and availability of insurance reimbursement.

Overall, the different areas in which the immunogenicity of transplanted islet cells can be tackled that we have discussed here, can help to direct current and future research and development work, to better formulate strategies to minimise or circumvent immune recognition and rejection in islet transplantation. These strategies will not only positively impact the lives of patients with complex T1D who tend to develop complications from conventional therapy, but hopefully be accessible by a wider group of diabetes patients in future.

## Author contributions

BXH: Conceptualization, Visualization, Writing – original draft, Writing – review & editing. AKKT: Conceptualization, Funding acquisition, Supervision, Writing – review & editing. NHJN: Conceptualization, Supervision, Writing – original draft, Writing – review & editing.
